# Patient Involvement to Promote Empathy in Preclinical Medical Students: Cross-Sectional Intervention Study

**DOI:** 10.2196/88184

**Published:** 2026-03-05

**Authors:** Rachel Winter, Jamanda Liddicott, Alice Delmonte, Cameron Dinnie, Amber Bennett-Weston, Mark Hamilton, Jeremy Howick

**Affiliations:** 1 Stoneygate Centre for Empathic Healthcare, Leicester Medical School, University of Leicester, George Davies Centre, University Road, Leicester, England, LE1 7RH, United Kingdom, 44 116-229-3770

**Keywords:** patient involvement, empathy, medical education, patient engagement, preclinical education, curriculum intervention

## Abstract

**Background:**

Despite increasing patient involvement in medical education, research has predominantly focused on involvement in small-group teaching. This study explored what it means to actively and meaningfully involve patients in large-group, lecture-based teaching while avoiding historical paternalistic approaches.

**Objective:**

This study aimed to describe the design, implementation, and evaluation of a novel curriculum component involving patients in early-year biomedical, clinical, and social science teaching to promote empathy in medical students.

**Methods:**

A 6-step approach to curriculum development was applied to guide the design of this curriculum component, enhancing the existing curriculum by hosting real patients in lectures to add a genuine and authentic patient voice. The design process was supplemented by a coproduction workshop with patients, educators, and students. Patients were recruited to take part via local health care networks and the University of Leicester Patient and Carer Group. Nine modules in years 1 and 2 hosted patients in lectures across the 2023-2024 academic year. A student feedback questionnaire based on previous similar published studies was developed to assess engagement and achievement of learning outcomes.

**Results:**

First- and second-year students (N=604) attended mandatory biomedical, clinical, and social science lectures hosting patients throughout the 2023-2024 academic year. In total, 65.6% (396/604) of students completed feedback questionnaires at the end of the year. Most students (340/391, 87%) reported that including patients in lectures increased their feelings of empathy, and 77.5% (307/396) reported that their inclusion improved their engagement with learning.

**Conclusions:**

The novel inclusion of real patients and their stories in biomedical, clinical, and social science lecture-based teaching has the potential to improve student learning and enhance feelings of empathy toward patients. Our findings are reproducible and transferable, and the intervention was well received by students.

## Introduction

### Background

Clinical empathy has multiple benefits for patient care [1] and practitioner health and well-being [2]. Empathy is usually considered to be a multidimensional construct incorporating affective, cognitive, behavioral, and moral components [3]. A recent systematic review of empathy definitions identified 6 components of empathy: exploring, understanding, shared understanding, feeling, therapeutic action, and maintaining boundaries [4]. These components overlap with the 2002 definition of clinical empathy in health care by Mercer and Reynolds [5]: the ability to understand the patient’s situation, perspective, and feelings; communicate that understanding to them; and act on it in a helpful and therapeutic way.

Empathy is central to health care education [6] and is recognized internationally as a professional competency expected of medical students and physicians [6-8]. However, despite its importance, empathy appears to decline during medical school [9,10]. While there is no consensus on the most effective means of teaching empathy [11-14], a systematic review of 26 trials demonstrated that empathy can be taught [14] and that specific sustainable interventions integrated into the curriculum are likely to be most effective [13].

There is current evidence suggesting that empathy can decline in students during the transition from preclinical to clinical-based education [9,10]. Some studies report that an undue focus on biomedical knowledge [15,16] often detached from the lived patient experience in the preclinical years contributes to this [16]. Other studies suggest that involving patients and their stories more consistently could help boost empathy [17-19], promote human attributes [18], support a focus on the psychosocial aspects of illness [19], encourage active learning [20], and enhance knowledge construction and clinical reasoning [21]. Despite the potential to benefit medical students, patients and their stories are rarely involved to support learning in the biomedical science–focused curriculum.

### Study Objectives

Historically, patients have been used, often unethically, in lecture theaters as “passive props” to illustrate pathophysiology [22]. This is reflective of an outdated, paternalistic era of medicine [22,23]. With a move toward patient partnerships [24], patients’ roles in medical education have become increasingly active [22], with patients and educators keen to find new, innovative ways to support patient involvement in medical education [25]. To address the lack of patient participation in the biomedical science curriculum, we developed, implemented, and evaluated a curriculum component that aims to promote empathy by involving real patients in lecture-style teaching for biomedical- and clinical-focused modules.

Our research questions were as follows: (1) What benefits to learning and engagement do students report when patients contribute to biomedical and clinical science lectures? (2) Does the inclusion of real patients and their stories in lectures during the early-year biomedical, clinical, and social science curriculum have the potential to enhance student empathy?

## Methods

We followed the six-step approach to curriculum development and implementation in medical education by Kern et al [26]: (1) problem identification and general needs assessment, (2) targeted needs assessment, (3) goals and objectives, (4) educational strategies, (5) implementation, and (6) evaluation and feedback. We refined our curriculum component by eliciting the ideas and opinions of medical educators, students, patients, and carers in a coproduction workshop.

### Problem Identification and General Needs Assessment

The General Medical Council emphasizes that medical students should cultivate a person-centered approach throughout their training, recognizing that placing patients at the center of care is fundamental to safe and ethical practice [27]. Educators and curriculum developers advocate for the integration of the biopsychosocial model (a framework for understanding health and illness that highlights interactions among biological, psychological, and social factors) throughout the early years, not as an adjunct but woven into teaching. This encourages students to learn a holistic approach to patient care [28] and holistic reasoning alongside biomedical sciences [29]. Medical students highlight that early exposure to psychosocial narratives enhances empathy, communication, and understanding of illness, particularly in the context of chronic and complex conditions [30]. Finally, patients recognize the importance of using a biopsychosocial lens from the outset to support person-centered values in future health care professionals [31].

### Targeted Needs Assessment

Leicester Medical School (LMS) describes its curriculum as integrated, patient focused, and with “teaching and learning based around patients and their needs” [32]. However, with notable exceptions [33], an audit of the curriculum (unpublished) revealed that most year 1 and 2 modules are biomedical and clinical science–based and use the lecture theater setting, which is devoid of exposure to real patients.

### Curriculum Development

#### Goals and Objectives

The goal of this intervention was to develop and deliver a curriculum component and evaluate the impact of involving real patients in biomedical and clinical science lectures on student empathy. We anticipated that having real patients contribute to lectures would support students in achieving the following intended learning outcomes:

- Being able to connect clinical science concepts to real-world patient presentations and scenarios
- Acquiring a deeper appreciation of the psychological and social impact of illness and disease on patients’ lives
- Starting to recognize the benefits of developing an empathic approach to patient care through reflection and discussion of patient experiences

#### Educational Strategy

Lectures have been, and continue to be, an efficient and standardized opportunity to deliver knowledge to large groups of students. However, this strategy is often teacher focused and can fail to engage students in active learning [34]. Active learning is student focused and aims to engage students by providing opportunities to interact, think, and discuss what they have been exposed to [35]. While the preclinical years at medical school tend to be focused on knowledge acquisition in the basic sciences, they are also an opportunity to provide formative lifelong learning and preparation for clinical practice [36]. Introducing real patients to lecture-based teaching of biomedical and clinical skills provides opportunities for students to interact and engage with their learning [37]. This educational strategy, added to the existing curriculum as a new component, can help students meet the required learning objectives, including knowledge acquisition; better understand patient perspectives; and help them prepare for future practice [35-37].

#### Coproduction Workshop

Coproduction involves key stakeholders working together, sharing power and responsibility from the beginning of a project [38,39]. A coproduction workshop was convened at the start of this project to engage stakeholders and improve the quality and relevance of this initiative [40], using an approach that is becoming increasingly popular [39]. The workshop included 3 medical educators (all module leads in the first 2 years of medical school [phase 1] at LMS), the project lead (RW) and another author (AB-W), 2 community patient representatives from the University of Leicester Patient and Carer Group, and 2 volunteer medical students. Students, educators, and patients broadly agreed that the desired outcomes of involving patients in biomedical science teaching were to (1) link theory to practice (and patient presentations) and (2) develop students’ understanding of the psychosocial impact of disability and/or disease. There was much discussion regarding possible approaches to involving patients in lectures, with the patient representatives keen to see their stories and experience interwoven and clearly relevant to the particular topic of the lecture. Student representatives felt that the opportunity to ask patients questions in lectures would be helpful, although there was some concern from educators about how this would be managed, particularly if questions were not appropriate or if there were no questions when students were prompted to ask them. The use of interactive tools in lectures for students to ask questions anonymously, for example, was discussed as a possible solution.

Educators and patient representatives discussed ways in which patient involvement in lectures could be evaluated, for example, through student feedback. Student representatives within the workshop identified possible challenges here, with frequent requests to provide feedback possibly being burdensome for students. However, patients felt that some form of feedback and evaluation of their involvement in lectures would be helpful, and it was agreed by educators that this should be a mandatory part of the intervention. Strategies discussed included a short debrief with patients and educators immediately after the lecture and specific student groups (eg, student societies with an interest in education) being asked to provide feedback following lectures hosting patients.

All workshop participants raised potential challenges. Educators were concerned about managing students who failed to engage or connect with learning and avoiding disruptive or distracting behavior. There was also concern that nonclinical educators may not feel confident about hosting patients in their lectures. Patient representatives felt that patients may be reluctant to travel to the university to engage for a short time (perhaps only 10-15 minutes). Patient representatives put forward concerns about potential consequences for patients involved and the need for a clear brief and clearly defined role for patients about what their involvement would mean. The group acknowledged that the intervention could cause patient anxiety about revisiting negative experiences, fear of judgment from students, and concern about confidentiality [22].

On the basis of findings from the coproduction workshop, modifications were made to the original intervention. Patient and educator representatives agreed that preparation for patients, carers, and educators would be key to ensure confidence, with the following measures put in place: patients should be provided with an overview of the lecture topic before taking part, patients should be given a clear idea of how their story is relevant and what aspects of their story would be most useful, patients should be given enough time to ensure that they are well prepared, and patients should be offered a debrief immediately after the event and a follow-up meeting several weeks after to provide any relevant student feedback. In addition, patients would be compensated for their time and support. Patient representatives in the workshop strongly felt that involving experienced patients would likely help lower the risk of experiencing discomfort or trauma if students appeared disengaged or engaged in unprofessional conduct. A standard operating procedure was developed to support both patients and lecturers once involved.

#### Curriculum Intervention

Permission to undertake this curriculum component was sought by internal stakeholders, including the head of the medical school and phase 1 (year 1 and 2) leads. Meetings were held with individual module leads to explain the intervention, encourage engagement, and offer support. Once a module lead agreed to take part, we worked to identify which lectures could host a patient and what type of patient or aspect of illness, treatment, or recovery would best fit. Patients were recruited through a variety of patient networks (including local, regional, and national patient groups; the University of Leicester Patient and Carer Group; and through clinical contacts at local primary and secondary care trusts). Patients who agreed to take part met with the lecturer to gain an understanding of the lecture structure and content and plan their contribution.

#### Curriculum Evaluation

The achievement of the intended learning outcomes was assessed by gathering student feedback at the end of the year. We developed an evaluation questionnaire consisting of 7 questions using a 5-point Likert scale and a free-text question. The questions were guided by those presented in a previous related study [19] and developed by 2 authors (RW and JL). The questions aimed to assess student perception of their engagement, learning, and satisfaction with lectures involving real patients. The questionnaire was distributed to students via an online platform. Free-text responses to the question “Are there any other comments you have about patients in lectures?” were analyzed to identify patterns through thematic analysis. Thematic analysis involves initial familiarization with the data, followed by coding, development of themes, and reporting of the findings [41]. There are concerns about the limitations of open-ended survey questions in supporting rigorous qualitative insights [42]. Data collected in this way may only consist of a few lines (or less) and may lack “attention to context and...conceptual richness” [43]. However, LaDonna et al [42] and others recognize that written survey responses can enhance findings, corroborate answers to closed-ended questions, and inspire new avenues of research. They propose strategies to guide free-text analysis and provide more meaningful findings, which were used to inform the analysis of data in this study.

### Ethical Considerations

Ethics approval for this evaluation was obtained from the Medicine and Biological Sciences Research Ethics Committee at the University of Leicester (42549-rw205-ls:medicine). There was no potential harm to participants. Any personal information that could directly identify participants will be removed or coded before study data are shared. Despite these measures, anonymity was not guaranteed. All patients were given a participant information leaflet and asked to give their consent for the lecture to be filmed. Participants were not offered compensation.

## Results

From the 18 modules in phase 1, a total of 11 (61.1%) module leads agreed to take part in the initiative. However, 18.2% (2/11) of these modules did not host patients (1 due to staff sickness and 1 because no patient could be found). Of the 9 modules (accounting for 9/18, 50% of phase 1 modules) that did host patients, 4 were first-year modules and 5 were second-year modules. Table 1 provides an overview of the modules included and details on the patients who were hosted in the lectures.

**Table 1.** Overview of the modules taking part in the initiative and patients hosted in the lectures.

Name of module	Year	Semester	Name of lecture	Patient presentation
Medical Cell Biology and Genetics	1	1	Genotype, phenotype, and inheritance	Cystic fibrosis
Population and Social Science	1	1	Long-term conditions	Multiple sclerosis
Cardiovascular System	1	2	Congenital heart disease	Congenital heart disease (AVSD^a^)
Musculoskeletal System	1	2	Back pain	Chronic back pain
Reproductive System	2	3	Menopause	Menopause
Respiratory System	2	3	Asthma	Asthma
Urinary System	2	3	Chronic kidney disease and dialysis	Peritoneal dialysis
Clinical Pharmacology Therapeutics and Principles of Prescribing	2	4	Epilepsy	Epilepsy (patient DNA)
^b^Integration for Clinical Application	2	4	Breast disease	Breast cancer

a AVSD: atrioventricular septal defect.

**Figure 1.**
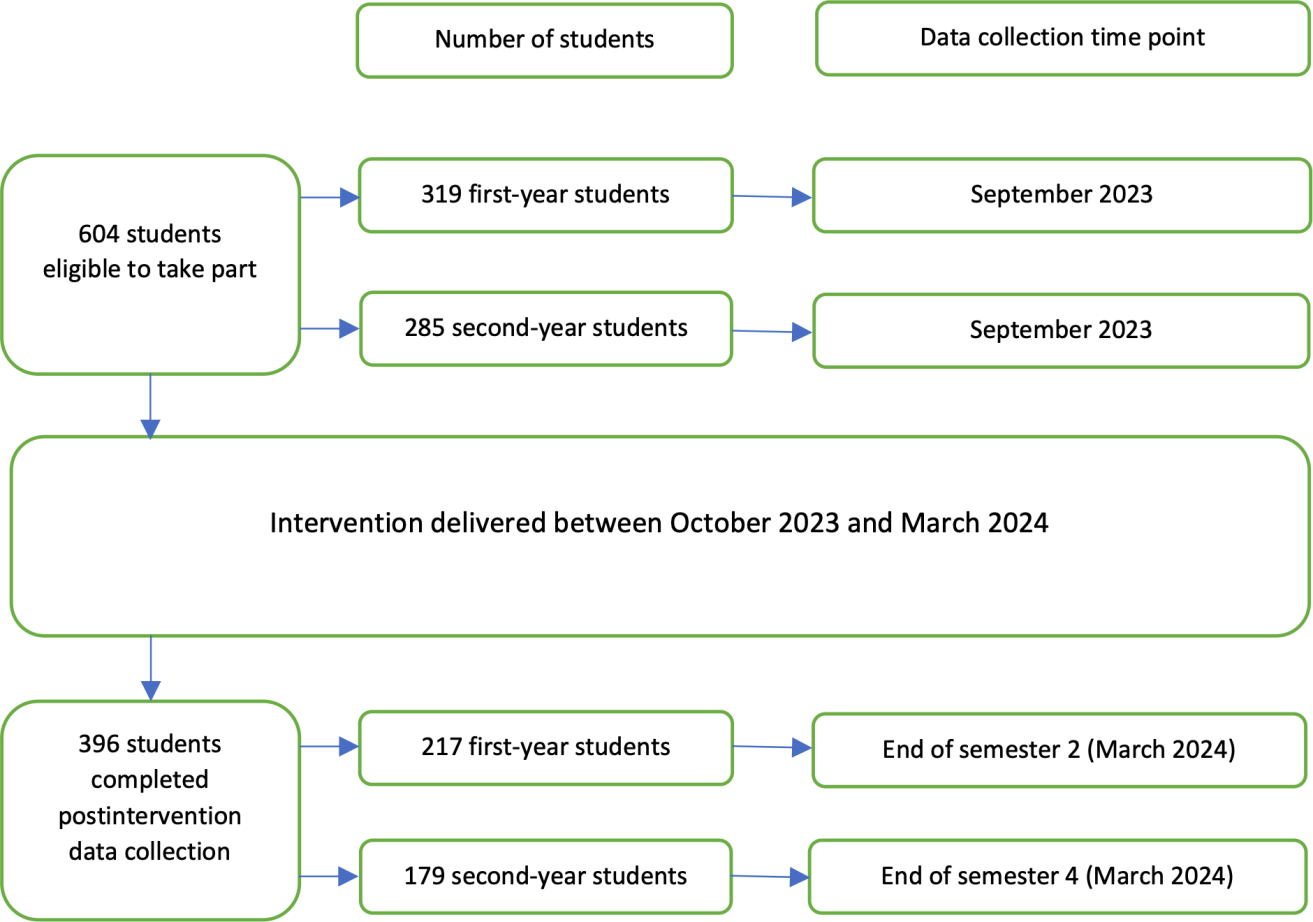
Overview of participant information and data collection time points.

At the end of the year, all students were invited to complete the feedback questionnaire. A total of 396 students completed the evaluation (396/604, 65.6% response rate), with 217 (54.8%) first-year students and 179 (45.2%) second-year students taking part. In total, 1.5% (6/396) of the students did not answer all 6 evaluation questions but were included in the evaluation data. Figure 1 provides an overview of participant demographics and data collection time points.

**Figure 2.**
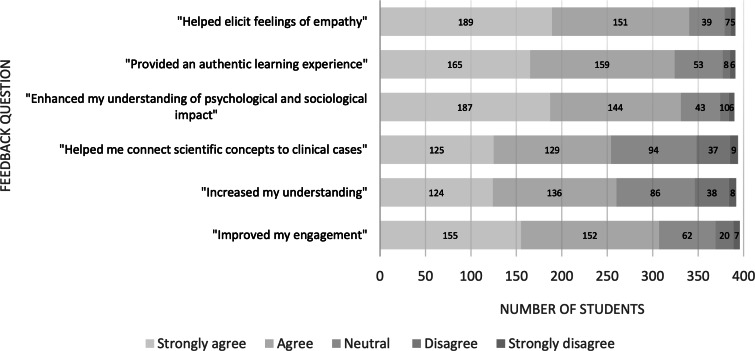
Overview of student feedback following participation.

Following the intervention, 87.0% (340/391) of the students agreed or strongly agreed that it helped elicit feelings of empathy. Most students (331/390, 84.9%) agreed or strongly agreed that including patients in lectures helped enhance their understanding of the psychological and social impact of a disease. In total, 77.5% (307/396) of the students agreed or strongly agreed that having patients involved in lectures improved their engagement. Figure 2 provides an overview of the findings. Nearly three-quarters of the students (281/396, 71.0%) agreed or strongly agreed that they would like to see more patients involved in lectures in the future.

In total, 8.8% (35/396) of the students provided free-text comments, and 2 themes emerged from these data. The first was “patients in lectures enhance understanding” (the most dominant theme). Students commented on how real stories helped them connect the subject matter to patient lives and described a deeper understanding and awareness when patients presented in lectures. In the second theme, “patients in lectures can present limitations,” students described some potential challenges of patients in lectures, including worrying that patients may feel uncomfortable and a lack of time in lectures to really engage with patients. Table 2 provides an overview of each theme with examples of supporting comments.

**Table 2.** Summary of free-text comments from students.

Theme	Description	Examples of supporting comments
Patients in lectures enhance understanding	Students described how patients attending their lectures enhanced their learning by helping them connect the subject matter to real stories. They described a deeper sense of understanding through an awareness of the psychosocial impact of illness. Students felt that the presence of patients in lectures created a more personal learning experience and helped foster empathy.	“They are all really lovely and they help me understand their condition and how it impacts them better.” “It’s so helpful and helps deepen understanding and empathy.” “Can we have more patients please in lectures when talking about clinical conditions. It makes them more engaging and more personal.” “Diseases are more memorable when I can associate it to a patient.” “I think having a patient come in revitalised my passion for medicine and reminded me why I came to medical school. It also helped me to better understand the physiology and pathology and helped me remember it better.” “They were good in telling us their stories and helping us understand the impacts on their lives and patients lives in general.”
Patients in lectures can have limitations	Some students found patients presenting in lectures to be less helpful, feeling that their input would be better in small-group sessions or on clinical placement. Some students worried that patients looked uncomfortable at times and seemed a little unsure of their role, possibly leading to students feeling unsure of how to engage. Students also felt at times that the experience could have been better; for example, they reported that patients were at times difficult to hear and some lectures ran over the allotted time when patients attended, leaving students finding it harder to concentrate for longer.	“Some have been really clear, but some seem nervous and waffle a bit too much.” “I feel like a video entry from the patients would have the same effect. I also found that often the patients looked like they felt uncomfortable and the students were reluctant to ask personal questions in front of the lecture theatre.” “Their stories are interesting but I don’t feel like I learn anything new than from the case studies and placements.” “Lectures overrun when we have patients and it’s harder to concentrate.” “It can be hard to engage in the whole lecture theatre. It might be more helpful to have them in smaller groups, but I understand it may be more difficult.” “We should be able to ask them our own questions.”

## Discussion

### Principal Findings

To the best of our knowledge, this paper is among the first to describe the design, implementation, and evaluation of a wide-reaching educational strategy that integrates real patients to promote empathy across the biomedical, clinical, and social science components of the medical school curriculum. We recognize that similar initiatives may be underway elsewhere and would welcome engagement with others working in this area. Students overwhelmingly reported that the inclusion of patients in science-based lectures helped elicit feelings of empathy and agreed that the intervention benefitted their engagement with teaching and learning.

Overall, our findings broadly support those of previous studies in this area. A systematic review of 49 studies reported that patient involvement in education in medical school can improve student understanding of person-centered care [44] and, therefore, empathy [45]. However, none of the studies included in this review described the introduction of real patients in lecture-based pathophysiology teaching. Other studies have identified that students value interactions with patients, reporting finding real patient encounters to be authentic and instructive [46], and that patients can help link theory with reality, enhance learning [46], and improve learning satisfaction [47]. Our evaluation adds to a growing body of evidence on the advantages of including real patients across the spectrum of medical education and in more innovative ways [19,47]. Specifically, it is rare to introduce real patients in lectures focused primarily on pathophysiology teaching.

### Strengths and Limitations

A strength of this evaluation is that it describes the development and delivery of a wide-reaching curriculum component. Our response rate to the postintervention survey was high, especially given the frequent requests that medical students receive to complete evaluations or participate in research [48,49]. Our findings identify that this addition to the curriculum is feasible and sustainable. There are some limitations to this evaluation. First, concerns were raised about the generalizability of our findings. This was a single-site evaluation for first- and second-year medical students. There are additional resources required to develop and implement this strategy across multiple sites, including the potential additional work required from faculty. To mitigate this, medical schools are required to promote a person-centered approach to health care throughout their programs [27], so lecturers already often include videos or vignettes (which must be identified and integrated into the lecture). There is also a cost associated with setting up the system of including patients in the lecture theaters. Our comprehensive standard operating procedure minimizes the set up and organizational resources required for future educators who wish to implement our curriculum component. We also note that our medical school has a patient involvement group who were happy to contribute to the delivery of this initiative. In addition, we worked with primary care colleagues at LMS to identify and recruit patients in general practice. Many medical schools have similar patient groups and networks that could be similarly helpful. Second, it was beyond the scope of this evaluation to formally explore patient experiences; however, we acknowledge that the patient experience does need exploring. We are currently conducting research to better understand the patient perspective when presenting in lectures, including any potential harms. A third limitation was that our evaluation did not describe the 4 types of evaluation by Kirkpatrick and Kirkpatrick [50], whose outcomes of hierarchy evaluate training methods at four levels: (1) reaction of the learner, (2) the degree to which the learning takes place, (3) how well the learning is assimilated into the learner’s practice or behavior, and (4) the degree to which the learning outcomes are met as a result of the training. We did not assess actual learning or changes to behavior or practice as a result of the intervention. Finally, while lectures are mandatory at LMS, there is no record of attendance kept, and it was not possible to identify whether students who completed the evaluation attended all or just some of the lectures hosting patients.

Involving patients in early-year teaching of biomedical and clinical science offers students a meaningful and motivating way to connect theoretical knowledge with real-world clinical practice. Patients from primary care settings provide ideal contexts for this intervention, offering a diverse range of experiences with conditions, chronic disease management, and preventative care. These real-life contexts not only illustrate the application of science but highlight the complexity and continuity of health care. Students will also begin to develop a broader understanding of the health care system, including social determinants of health and the realities of delivering care in community settings, right from the start of their training. There are inherent challenges, as already discussed, with inviting patients to attend lectures. However, there are many potential benefits to students in terms of fostering empathy toward patients, enhancing engagement with learning, and increasing awareness of the psychological and social aspects of health and illness. A future focus on the long-term impact on empathy and whether this intervention can help halt or reverse the documented decline in medical student empathy [9,10] during training is needed.

### Conclusions

Integrating real patients and their stories into biomedical and clinical science lecture-based teaching is a novel application. The introduction to real patients throughout the early-year biomedical, clinical, and social science curriculum was well received by students. It can support engagement with learning and promote feelings of empathy in students, with the potential to mitigate the risk of decline in empathy among medical students.

## Abbreviations

LMS: Leicester Medical School
